# *Moringa oleifera* Lam and its Therapeutic Effects in Immune Disorders

**DOI:** 10.3389/fphar.2020.566783

**Published:** 2020-12-17

**Authors:** Xiao Xiao, Jue Wang, Chen Meng, Weibo Liang, Tao Wang, Bin Zhou, Yanyun Wang, Xiaolei Luo, Linbo Gao, Lin Zhang

**Affiliations:** ^1^ Department of Obstetrics and Gynecology, West China Second University Hospital of Sichuan University and the Key Laboratory of Birth Defects and Related Diseases of Women and Children, Ministry of Education, Sichuan University, Chengdu, China; ^2^ Laboratory of Molecular Translational Medicine, Center for Translational Medicine, Key Laboratory of Birth Defects and Related Diseases of Women and Children (Sichuan University), Ministry of Education, West China Second University Hospital, Sichuan University, Chengdu, China; ^3^ Department of Forensic Genetics, West China School of Basic Medical Sciences & Forensic Medicine, Sichuan University, Chengdu, China; ^4^ Department of Geriatrics, Chengdu Second People’s Hospital, Chengdu, China

**Keywords:** *Moringa oleifera*, infection, chronic inflammation, physicochemical irritation, autoimmune disorders

## Abstract

*Moringa oleifera* Lam., a plant native to tropical forests of India, is characterized by its versatile application as a food additive and supplement therapy. Accumulating evidence shows that *Moringa* plays a critical role in immune-related diseases. In this review, we cover the history, constituents, edibility, and general medicinal value of Moringa. The effects of Moringa in treating immune disorders are discussed in detail. Moringa can not only eliminate pathogens, including bacteria, fungi, viruses, and parasites, but also inhibit chronic inflammation, such as asthma, ulcerative colitis, and metabolic diseases. Additionally, Moringa can attenuate physical and chemical irritation-induced immune disorders, such as metal intoxication, drug side effects, or even the adverse effect of food additives. Autoimmune diseases, like rheumatoid arthritis, atopic dermatitis, and multiple sclerosis, can also be inhibited by Moringa. Collectively, Moringa, with its multiple immune regulatory bioactivities and few side effects, has a marked potential to treat immune disorders.

## Introduction

*Moringa oleifera* Lam (MO), a frost and drought resistant plant of the monogeneric family Moringaceae, a native plant of tropical forests of India, is characterized by its versatile applications as a food additive and supplement therapy (Anwar et al., 2007). MO is suitable for food application because of its abundant nutritional ingredients, such as essential amino acids, oleic acids, vitamins, and minerals. MO is recognized for its medicinal uses, such as treating various infections, modulating the immune system, and displaying anti-oxidant, anti-diabetic, or anti-tumor effects (Dhakad et al., 2019).

Moringa tree leaves were mostly used for cattle feed in ancient times (Sun et al., 2017), but were gradually started to be used in the human diet to maintain mental and skin health (Anwar et al., 2007). With its growing popularity, different parts of MO, such as roots, seeds, and pods, were recognized as nutritious and medically valuable. Currently, MO is widely used in food ingredients, nutraceuticals, and medications and has been termed a “Miracle tree” (Dhakad et al., 2019).

## Bioactive Constituents and General Function of *Moringa oleifera*

The bioactive constituents of MO have been identified in almost all parts of the plant (Liang et al., 2019). The specific constituents isolated from MO mainly (detailed in Supplementary Table S1) include flavanoids (mainly distributed in the leaves), glucosinolate and isothiocyanate (mainly distributed in the leaves), phenolic acid (all distributed in the leaves), alkaloids and sterols (distributed in the leaves, roots, and seeds), and terpene (all distributed in the pods) (Anwar et al., 2007; Bichi, 2013; Baldisserotto et al., 2018; Dhakad et al., 2019). The constituents of the leaves and seeds were most frequently reported. Based on the phytochemical analysis, phenols and alkaloids are more abundant in the leaves than in the seeds, while flavonoids, saponins, and anthocyanins are more abundant in the seeds (Gupta et al., 2018). Besides, other kinds of nutrients are present in high levels in the processed products of MO, including a number of fatty acids derived from the seed oil (Leone et al., 2016), various kinds of minerals from the dried leaf powder (Witt, 2014), and high-quality carbohydrates from refined gum exudates (Kar et al., 2013; Gupta et al., 2018).

The addition of a small amount of MO is reported to significantly improve the nutritional value of food such as bread, yoghurt, cheese, and soup (Williams, 2013; Stadtlander and Becker, 2017). The diverse parts of MO have been processed into many food products in more than eighty countries, to improve mineral and vitamin deficiencies (Ali et al., 2017). Moreover, few side effects have been reported for the use of MO (Bichi, 2013; Palada et al., 2017; Dhakad et al., 2019).

In terms of its therapeutic properties, the constituents isolated from the seeds and leaves of MO are reported to function in approximately 80 diseases (Mahmood et al., 2010), which can be mainly categorized as oxidative stress, glucose metabolism disorders, tumors, organ injury, and immune-related diseases (Anwar et al., 2007; Dhakad et al., 2019). MO contains more than 40 natural antioxidant compounds and is well-known for its effect on eliminating free radicals (Pakade et al., 2013). For example, isoquercetin is recorded to have the highest antioxidative activity and exhibits a ROS inhibitory effect by increasing the expression of antioxidant enzymes, such as superoxide dismutase (SOD), glutathione peroxidase (GPx), and catalase (Vongsak et al., 2013, 2015; Ratchanee, 2014). In addition, the application of MO leaf powder can maintain malondialdehyde (MDA) levels and the ferric reducing ability of human plasma (Doerr, 2005; Ngamukote et al., 2016). MO has shown outstanding hypoglycemic activity in various diabetic animal models, or in human volunteers, because it can not only stimulate insulin secretion from pancreatic β-cells, but also directly reduce blood glucose by reacting with anti-insulin antibodies (Mahmood et al., 2010; Tende et al., 2011; Villarruel-López et al., 2018). MO also exhibits antitumor properties, including cytotoxic, antiproliferative, chemoprotective, and anti-inflammatory activities in diverse types of tumors (Guevara et al., 1999; Biswas et al., 2012). Moreover, MO is also reported to protect organs from injury. MO not solely stabilizes hypotensive activity to protect the cardiovascular system via its fully acetylated glycosides, but also has a calcium antagonist effect, a lipid removal function, and diuretic activity (Faizi et al., 1998; Sana et al., 2015). Furthermore, MO showed antispasmodic, antiulcer, and hepatoprotective effects in treating diarrhea, gastrointestinal motility disorder, and fatty liver disease (Hamza, 2010; Kumar et al., 2010; Das et al., 2011). One of the most valuable effects of MO is its immune-related functions, which have been reported recently years, which are involved in many immune disorders and possesses significant value in translational medicine. In the present review, we introduce and discuss the immune-related functions of MO.

## Anti-Infectious Activity of *Moringa oleifera*

MO possesses a number of activities against infectious diseases. All parts of the plant can be made into various formulations against bacteria, fungi, viruses, and parasites.

Bioactive components of natural medicinal herbs, including MO, exert the anti-infectious effects against pathogens. Benzyl isothiocyanate, extracted from the seeds of MO, can significantly reduce the pathogenicity of bacteria by inhibiting bacterial conjugation (Padla et al., 2012). A leaf extract containing silver and niaziminin, or flowers containing kaempferol, rhamnetin, and isoquercitrin (Dubey and Gupta, 1978; Das et al., 2013; Vongsak et al., 2013; Rajendran et al., 2014; Paikra et al., 2017), exerted a direct beneficial effect in the elimination of microbes. The anti-bacterial activity of MO is summarized in detail in Table 1. It is clear that MO has a relatively broad anti-microbial spectrum; however, it shows a slightly higher inhibitory effect against gram-negative bacteria. The other anti-infectious activities of MO are summarized in Table 1 as well. The leaves and seed appear to possess a broader spectrum of anti-microbial activity than the other parts of MO. It is acknowledged that bioactive components of MO have been fully researched, however, what is the corresponding relationship between different parts of MO and their effects to certain infectious diseases remains to be discussed.

**TABLE 1:** Anti-microorganism activity of *Moringa oleifera* Lam.

Categories	Species	Gram staining	Source of active components	Representive literatures (PMID, DOI)
Bacteria	Aeromonas caviae	G-negative	Leaf	21771453
	Bacillus cereus	G-positive	Seed	30810376
	Bacillus megaterium	G-positive	Bark	26109449
	Bacillus subtilis	G-positive	Leaf	30810361
	*Citrobacter freundii*	G-negative	Bark	25362592
	Enterobacter aerogenes	G-negative	Leaf and seed	26753836
	Enterococcus faecalis	G-positive	Leaf	27776261
	Escherichia coli	G-negative	Leaf, seed, pod	20602021, 26753836, 21605145, 25410525
	Klebsiella pneumonia	G-negative	Leaf, seed, pod	26753836, 25410525, 18686107
	Mycobacterium phlei	G-positive	Leaf	22556699
	Proteus mirabilis	G-negative	Seed	29142400
	Providencia stuartii	G-negative	Leaf	26753836
	Pseudomonas aeruginosa	G-negative	Leaf and seed	1921416, 26753836, 25410525
	Salmonella typhimurium	G-negative	Pod	25410525
	Serratia marcescens	G-negative	Leaf and seed	28792661, 20677603
	Staphylococcus aureus	G-positive	Leaf, seed, pod, and bark	1921416, 20602021, 21605145, 25410525, 25410525
	Staphylococcus epidermidis	G-positive	Pod	25410525
	Streptococcus pyogenes	G-positive	Leaf and seed	24205757
	Vibrio cholerae	G-negative	Leaf and flower	20602021, 26614991
	Vibrio mimicus	G-negative	Flower	26614991
	Vibro vulnificus	G-negative	Flower	26614991
	Yersinia enterocolitica	G-negative	Seed	DOI: 10.1016/j.jff. 2013.09.009
Fungus	Aspergillus flavus	-	Leaf and seed	25742976, 24022760, DOI: 10.1016/j.sajb. 2014.02.002
	Aspergillus Niger			
	Basidiobolus haptosporus		Leaf	8531930
	Basidiobolus ranarum			
	Candida albicans		Seed	22367027, 25410525, 31085448
	Cryptococcus neoformans		Leaf	DOI: 10.1016/j.sajb. 2014.02.002
	Epidermophyton floccosum		Leaf and seed	16406607, 23413749
	Microsporum canis		Leaf and seed	16406607, 23413749
	Penicillium aurantiogriseum		Leaf	24022760
	Penicillium citrinum			
	Penicillium digitatum			
	Penicillium expansum			
	Trichophyton mentagrophytes		Leaf and seed	16406607, 23413749, 8531930
	Trichophyton rubrum			
Parasite	Brugia malayi	-	Gum	31453658
	Cryptosporidium parvum		Leaf	31406404
	Dracunculiasis		Leaf	7758381
	Haemonchus contortus		Seed	30255045, 25054490
	Helminth		Seed	22546609
	Hymenolepis nana		Leaf	28382873
	Leishmania donovani		Flower	26669018
	Plasmodium		Seed	12064726
	Schistosomes		Flower	28994007
	Trypanosoma brucei		Aerial parts	24310210, 20359874, 10479771
Virus	Epstein-barr virus	-	Leaf and seed	9619112, 10209341, 24577932
	Equine herpes virus		Leaf	27393440
	Foot and mouth disease virus		Leaf	29175786, 27393440
	Hepatitis virus		Dry powder	23216691, 29214184
	Herpes simplex virus		Leaf	26814058, 19666102, 14638393
	Human immunodeficiency virus		Leaf	24096203, 29154059
	Infectious bursal disease virus		Leaf	DOI: 10.2478/s11535-013-0,276-8
	Rhinovirus		Leaf	29653740

PMID: PubMed Unique Identifier; DOI: Digital Object Identifier.

Mechanistically, one of the most effective ingredients of MO is the moringa coagulant protein (molecular weight approximately 13 kDa), which can purify polluted water, regulate its acid-base balance, and exert an antiseptic effect, even in a crude salt extract form (Anwar et al., 2007; Abdul Hamid et al., 2016; Gupta et al., 2018). The coagulant protein is able to flocculate microorganisms through its functions of adsorption and charge neutralization (Broin et al., 2002; Mulugeta and Fekadu, 2014). In addition, a group of amino acids found in MO can interact with metal ions. These amino acids, together with the absorbed metal ions, generate a negatively charged environment that consistently influences the survival of pathogens (Sharma et al., 2007; Obuseng et al., 2012; Matouq et al., 2015). Moreover, the ingredients in MO can be chemically functional. Kaempferol, a natural flavonoid extracted from MO, exhibits dose-dependent anti-microbial effect via disruption of the integrity of bacterial cell membrane (Poklar Ulrih et al., 2010; Rajendran et al., 2014). Isoquercitrin, another active ingredient of MO, can strongly inhibit viral gene expression by attenuating the activation of the nuclear factor kappa (NF-κB) signaling pathway (Vongsak et al., 2015).

Several reports have shared the function of MO in deliminating pathogens because the multiple effective components, including natural proteins and certain amino acids in MO, which can destroy or neutralize the microorganisms, modulate the microenvironment, and amplify the immunity. Although basic researches of MO were relatively common nowadays, clinical researches or applications based on the single component of MO were still infrequent. More clinical trials of MO should be applied when the safety of MO has been confirmed.

## *Moringa oleifera* and Its Effect on Chronic Inflammation

Chronic inflammation is involved in a number of disorders and is characterized by continuous expression of pro-inflammatory factors and long-lasting tissue damage. MOs possesses properties that act against chronic inflammation and its associated disorders.

An n-butanol extract of *Moringa* seeds could significantly improve the lung function parameters of guinea pigs with ovalbumin-induced airway inflammation (Mahajan et al., 2009; Mahajan and Mehta, 2011). The number of immunological cells, particularly neutrophils and eosinophils, was dramatically decreased in serum or in bronchoalveolar lavaged fluid when the extract was applied. This anti-inflammatory effect was also confirmed using lung tissue histopathology. The active ingredient of the extract, subsequently proved to be β-sitosterol, was believed to function by modulating the balance of Th1/Th2 cytokines. However, few studies have focused on the response of immunocytes to MO. Kooltheat et al. found that MO could eliminate the production of monocyte-derived macrophage factors, like tumor necrosis factor alpha (TNF-α), interleukin (IL)-6 and IL-8 (Kooltheat et al., 2014, 2017). Notably, the decrease in these factors was evident at both mRNA and protein levels. Most of immune-related molecules were originated from immunocytes. Therefore, it is critical to study the effects of MO on immunocytes and immuno-microenviroment.

Ulcerative colitis (UC), a chronic intestinal disease characterized by bloody diarrhea, is a non-specific inflammatory disorder as well as a common precancerous lesion of colectoral cancer. Minaiyan et al. used a hydro-alcoholic extract of *Moringa* to treat experimental colitis in mice, and observed downregulation of a group of secreted inflammatory factors and an increase of both colon lengths and the expression of glutathione-S-Transferase Pi 1 (GSTP1), which is a detoxifying enzyme mediated by NFE2-Related Factor 2 (NRF2) (Kim et al., 2017). This effect was attributed to compounds of biophenols and flavonoids in MO in a dose-dependent manner (Minaiyan et al., 2014; Kim et al., 2017).

Chronic inflammation is also associated with metabolic disorders, such as non-alcoholic steatohepatitis (NASH) caused by hepatic lipid accumulation, and high-fat diet induced glucose intolerance. Almatrafi et al. measured the levels of hepatic cytokines of guinea pigs fed with no, low, or high MO diets^48^. They demonstrated that the expression of IL-1β, IL-10, and interferon gamma (IFN-γ) were the lowest in the high MO group, and no difference was found for IL-6, monocyte chemoattractant protein-1 (MCP-1) and TNFα cytokines among the groups. The authors inferred that quercetin and chlorogenic acid might contribute to the anti-inflammatory effect (Bamagous et al., 2018). A similar diet (containing a fermented *Moringa* extract contained) was applied to experimentally obese mice. The expression of pro-inflammatory cytokines was markedly decreased in their liver, epididymal adipose, and quadriceps muscle (Joung et al., 2017). Traditional medicine has obvious advantages for chronic diseases, therefore, more clinical trials should be performed to understand the regulation process of MO.

Symptomatic support and immunity improvement are currently the first-line approaches for the treatment of chronic inflammation, mainly because chronic inflammation is often acompanied by disorder of the immune microenvironment. MO, not only inhibiting the expression of a series of pro-inflammatory factors, but also contributing to the regulation of immune cells, provides options for the control of chronic inflammation.

Collectively, MO could attenuate the negative impact of the chronic inflammation mainly through inhibiting the expression of a series of pro-inflammatory factors.

## Physicochemical Irritation Induced Immune Disorders and *Moringa oleifera*

MO may also be used to treat immune disorders after physical or chemical irritation. Since ancient times, MO has been used to treat cuts, burns, and wounds (Rathi et al., 2006; Mahmood et al., 2010; Paikra et al., 2017). MO is capable of inducing a moderate inflammatory phase after injury, which is critical for the wound healing cascade, because it provides a suitable environment for the removal of harmful substances and tissue repair, prevents excessive leukocyte recruitment, and promotes the proliferation and migration of fibroblasts. Additionally, several studies have validated the central and peripheral analgesic effects of MO (Rao et al., 2008; Adedapo et al., 2015; Martínez-González et al., 2017; Paikra et al., 2017), not to mention its anti-infection property, which has been widely demonstrated. These studies provided scientific support for the use of MO by indigenous Philippines and Indians who collected MO to dress wounds (Nama, 2015; Palada et al., 2017). MO is advantaged to deal with acute disorders because it is easily accessible and that's why MO has saved many lives in developing countries.

Chemical irritation mainly refers to metal intoxication and drug side effects, which induce global or organic immune disorders and tissue damage.

Adeyemi et al. found that MO-based diets could protect against nickel-induced hepatotoxicity in rats, partially by attenuating the systemic inflammatory response (Adeyemi et al., 2017). In another study, an ethanolic extract of MO was applied to chromium-treated male rats. MO significantly reduced the levels of inflammatory markers and ameliorated the chromium effects on testicular local immunity (Sadek, 2014). Besides, several studies have reported that heavy metal ions, such as Cd (II), Pb (II), and Cu (II), can be removed using the bark and seeds of MO; therefore, in addition to its anti-oxidative effect, MO-based therapy is recommended as a valuable treatment for detoxication (Sharma et al., 2007; Obuseng et al., 2012; Reddy et al., 2012; Chatterjee et al., 2016).

Edeogu et al. explored the protective effect of MO seed oil against gentamicin-induced pro-inflammation in rats (Edeogu et al., 2019). They found that gentamicin prominently increased the content of IL-6, TNF-a, induced nitric oxide synthase (iNOS), and NF-κB in the kidney, while treatment with MO significantly decreased the levels of these inflammatory markers. Overdose administration of acetaminophen, commonly considered as one of the leading causes of acute kidney failure, resulted in a significant elevation of pro-inflammatory cytokines (IL-1β, IL-6, and TNF-α) and a reduction in anti-inflammatory cytokines (IL-10) in renal tissue: All of these inflammatory changes were reversed by treatment with an MO leaf extract (Adil et al., 2016). Similar effects were found in treatment of levofloxacin-induced hepatic toxicity, aspirin-induced gastric ulcer, and methotrexate-induced neurotoxicity (Akhtar and Ahmad, 1995; Verma et al., 2012; Famurewa et al., 2019; Farid and Hegazy, 2019).

Moreover, the toxicity of food additives is partially attributed to inflammatory injury. MO has been used to ameliorate nephrotoxicity induced by titanium dioxide nanoparticles (TiO_2_ NPs) (Kandeil et al., 2019). TiO_2_ NP-treated rats were fed with an MO leaf extract, and the expression of kidney 222 injury molecule 1 (KIM-1), NF-κB, TNF-α, and heat shock protein 70 (HSP-70) were markedly 223 decreased, while the expression of NRF-2 and heme oxygenase 1 (HO-1) were significantly 224 upregulated compared with those in control groups. Recently, Abd-Elhakim et al. showed that MO 225 might exert a protective effect against melamine- induced hemato-immunotoxic hazards (Abd-Elhakim 226 et al., 2018). In that study, melamine had a markedly adverse impact on the global hematological 227 system, while the application of MO not only attenuated the melamine-induced symptoms of anemia, 228 leukopenia, and innate and humoral immune disorders, but also restored hematological parameters, 229 including neutrophils, lymphocytes, serum IgG and nitric oxide (NO) levels.

Physicochemical irritation tend to cause acute stress response. Considering that MO is an inexpensive and easily available natural plant with few reported side effects, it is an advantageous choice for the treatment of such diseases. Besides, MO has good analgesic effects and can be used to counteract side-effects of some medicine, it may be a useful attempt to use it as a companion drug.

## Auto-Immune Disorders and *Moringa oleifera*

Rheumatoid arthritis (RA) is a typical auto-immune disorder, characterized by an increase in pro-inflammatory cytokines (including TNF-α, IL-6, and IL-1β) and inducible inflammation-related enzymes (such as cyclooxygenase and lipoxygenase) and a decrease in anti-inflammatory cytokines (as IL-4 and IL-10). Several studies have reported the efficacy of MO in alleviating joint inflammation associated with RA; however, the exact mechanism remains unknown (Mahajan et al., 2007; Padmini et al., 2016). Saleem and colleagues used complete Freund's adjuvant to establish an RA model in rats. In the model, treatment with a MO methanolic extract markedly reduced the serum concentration of C-reactive protein, prostaglandin E2, and TNFα, markedly downregulated the levels of NF-κB, prostaglandin E2 (PGE2), cyclooxygenase 2 (COX-2), and IL-1β, and significantly upregulated the mRNA levels of *I-κB*, *IL4*, and *IL-10*, and remarkably restored the histopathological indices and arthritic index in the joints (Saleem et al., 2019).

Atopic dermatitis (AD), a kind of chronic, inflammatory skin disease, belongs to another group of classic auto-immune disorders (Brunello, 2018). It is generally accepted that AD is typically accompanied by an extreme initiation of T-cells, elevated serum IgE levels, and the skin infiltration of dendritic cells and T cells (Yamura et al., 1981). Choi et al. used TNF-α and IFN-γ to induce AD in HaCaT cells (human keratinocytes), and applied a *Dermatophagoides farinae* extract to monitor AD in BALB/c mice (Choi et al., 2016). MO not only reduced the expression of pro-inflammatory cytokine-related mRNAs and the levels of mitogen-activated protein kinases (MAPKS*) in vitro*, but also improved the ear skin thickness and serum immunoglobulin levels *in vivo*. In addition, a decrease in retinoic acid-related orphan receptor γT (RORγT) levels was observed, which regulates the expression and development of Th17 cells). Levels of thymic stromal lymphopoietin (TSLP, which triggers dendritic cells and secretion of Th2 cytokine production) and mannose Receptor C-Type 1 (CD206, which is expressed in various immunological cells) were also reduced. These results strongly suggested the efficacy of MO as a supplement to treat patients with AD.

Similar effects were demonstrated in a model of multiple sclerosis. Galuppo et al. showed that MO could counteract the inflammatory cascade in an animal model of experimental autoimmune encephalomyelitis (EAE) (Galuppo et al., 2014). In that study, TNF-α was identified as one of the main targets of glucomoringin-isothiocyanate (GMG-ITC), a natural agent extracted from MO.

Tahiliani and colleagues revealed the therapeutic value of MO leaf extract in the regulation of hyperthyroidism, an auto-immune related disorder, by inhibiting triiodothyronine (T3) synthesis and release (Tahiliani and Kar, 2000). Monotherapies provide limited benefits in curing autoimmune diseases while natual drugs like MO are excellent alternatives. Unfortunately, scholars interested with auto-immune diseases have merely conducted experiments of MO on animals.

Autoimmune diseases are mainly associated with genetic factors, immunomodulation, viral infection and antigenic variations. With the exception of genetic factors, MO has been reported to have unique positive effects on the other three aspects. Most of related reports were focused on its value for organ-specific autoimmune diseases. However, MO is a medicinal plant with multiple active ingredients, which may have a better effect on systemic autoimmune diseases, such as systemic lupus erythematosus. More researches on MO in systemic autoimmune diseases are warranted.

## Conclusion and Future Perspectives

**FIGURE 1:**
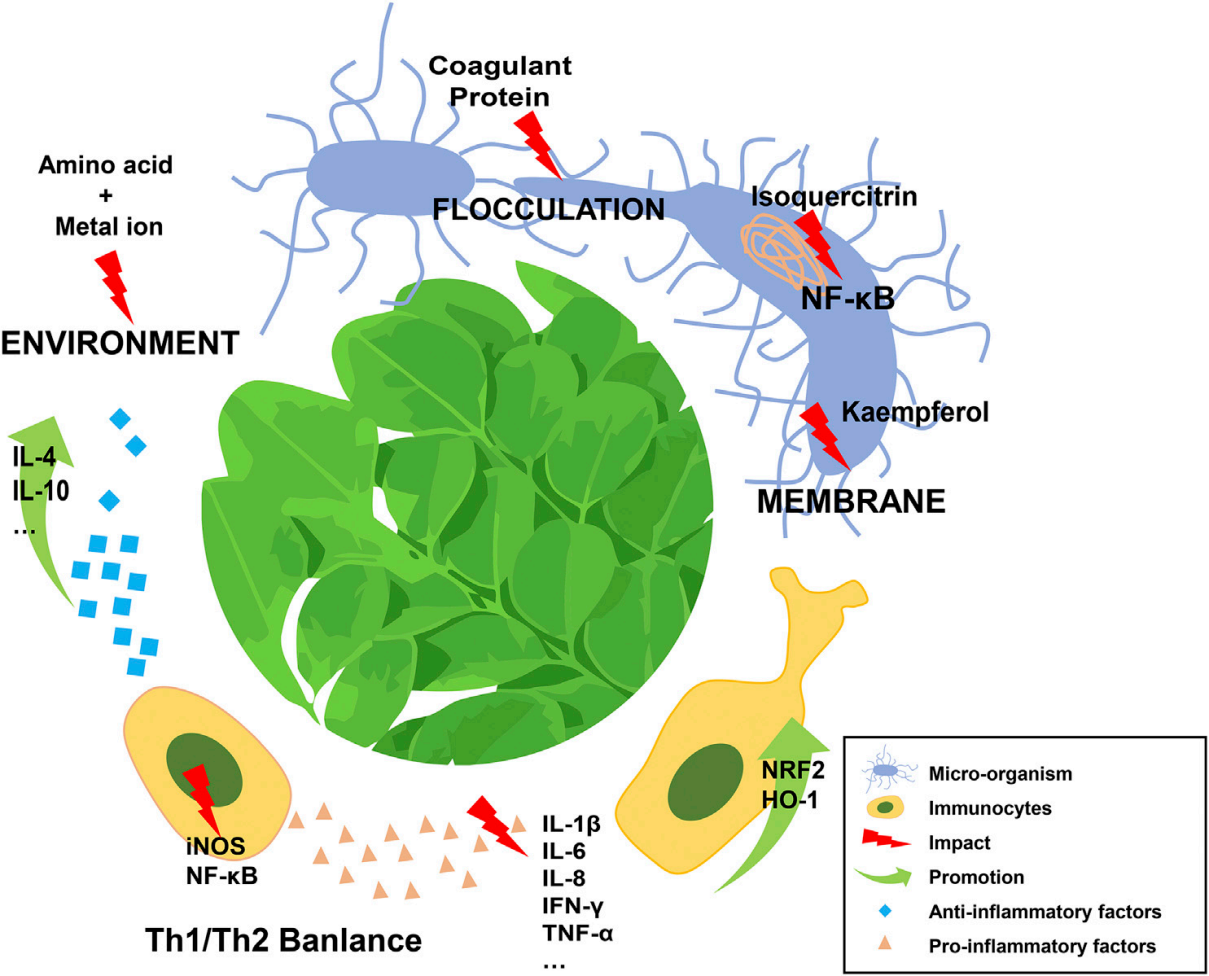
Schematic diagram of *Moringa oleigera* Lam in dealing with immune disorders.

Current research shows that MO exerts its multiple immune-related effects primarily through directly eliminating pathogens or modulating the balance of pro- and anti-inflammatory mediators released from various kinds of immune cells by regulating the activity of signaling pathways, such as the canonical NF-κB pathway (Figure 1). Significantly, the bioactivity of MO is dependent on its active ingredients, which are related to the different parts of this plant and extraction methods used. Notably, in some experiments, low-dose application of MO might have a better anti-inflammatory effect than higher doses (Ferreira et al., 2008; Almatrafi et al., 2017; Kapse et al., 2017), which suggested the necessity of identifying the appropriate dosage of MO before clinical application.

Research evidence has demonstrated the therapeutic value of MO to treat immune disorders; however, a few problems still remain to be solved. For example, will the active ingredients extracted from different parts of MO interact to invalidate its effects? Are there any side-effect remaining to be discovered? Are there other molecular mechanisms underlying MO’s immune-regulatory function?

Immune disorders, whether resulting from infection or inflammation, might have severe consequences. MO, with few reported side effects, has a long history for curing diseases and is an inexpensive and credible natural medicine. More importantly, it can precisely modulate the immune balance because of its moderate and comprehensive bioactivities. Despite its huge potential to cure immune disorders, further quantitative and mechanistic research should be undertaken before MO can be developed into clinical applications.

MO is most popular in east and southeast asian countries according to the current reports. It is also used in central america and other tropical countries because it is originated from tropical regions. The total extract of MO was cheap and effective which has saved a large number of patients in the less developed area of the world. However, as all the other natural medicine, it is almost impossible to be widely used in clinical until the risk of MO has been thoroughly understood. Unfortunately, there has been little research conducted specifically on the side-effects of MO, and further evidence is needed in the future to confirm the safety of MO.

## Author Contributions

LG and LZ designed the manuscript. XX and JW wrote the manuscript with essential contribution of CM, WL, TW, BZ, YW, XL, LG, and LZ. XX produced the figure.

## Funding

This work was supported by the National Natural Science Foundation of China (No. 32071182), the Fundamental Research Funds for the Central Universities (No. 2018SCUH0053), the Science and Technology Major Projects of Sichuan Province of China (No. 2017SZDZX0013), the Science and Technology Support Program of Sichuan Province of China (No. 2016SZ0013), and the National Natural Science Foundation of China (No. 81372377).

## Conflict of Interest

The authors declare that the research was conducted in the absence of any commercial or financial relationships that could be construed as a potential conflict of interest.

## Abbreviations

MO, *Moringa oleifera* Lam.; SOD, superoxide dismutase; GPx, glutathione peroxidase; MDA, malondialdehyde; NF-κB, nuclear factor kappa; TNF-α, tumor necrosis factor alpha; IL, interleukin; UC, ulcerative colitis; GSTP1, glutathione-S-transferase Pi 1; NRF2, NFE2-related factor 2; NASH, non-alcoholic steatohepatitis; IFN-γ, interferon gamma; MCP-1, monocyte chemoattractant protein-1; iNOS, nitric oxide synthase; TiO2 NPs, titanium dioxide nanoparticles; HSP-70, heat shock protein 70; KIM-1, kidney injury molecule 1; HO-1, heme oxygenase 1; NO, nitric oxide; RA, rheumatoid arthritis; PGE-2, prostaglandin E2; COX-2, cyclooxygenase 2; I-κB, inhibitor of nuclear factor kappa-B kinase; AD, atopic dermatitis; MAPKS, mitogen-activated protein kinases; RORγT, retinoic acid-related orphan receptor γT; TSLP, thymic stromal lymphopoietin; EAE, experimental autoimmune encephalomyelitis; GMG-ITC, glucomoringin-isothiocyanate; T3, triiodothyronine.
