# Supplementary material for: Moringa oleifera Lam and its Therapeutic Effects in Immune Disorders
Source: Front Pharmacol. 2020 Dec 17;11:566783. doi: 10.3389/fphar.2020.566783 (PMC7773658; doi:10.3389/fphar.2020.566783)
Supplement: Supplementary file 1 [file table1.docx]

Supplementary Material

| Supplementary table. Plant Parts, extraction methods, and common ingredients of MO Extracts | | |
| --- | --- | --- |
| Extract parts of MO | Extract methods | Common bioactive ingredients of MO |
| Leaf | Aqueous extracts | Flavanoids;  Glucosinolate;  Isothiocyanate;  Phenolic acid;  Terpene;  Alkaloid;  Sterol;  Oleic acid;  Nutritional composition; etc. |
|  | Acetone extracts |  |
|  | Butanoic extracts |  |
|  | Dried or wet leaf powder |  |
|  | Ethanolic extracts |  |
|  | Ethyl acetate extracts |  |
|  | Fresh juice or decoction |  |
|  | Hexane extracts |  |
|  | Methanol extracts |  |
| Seed | Aqueous extracts |  |
|  | Ethanol extracts |  |
|  | Ethyl acetate extracts of seeds |  |
|  | Methanolic extracts |  |
|  | Seed oil |  |
|  | Seed powder |  |
|  | Seed coat |  |
|  | Salt solution of seeds extract |  |
| Root & stem bark | Aqueous extracts |  |
|  | Ethanolic extracts |  |
|  | Juice of root bark |  |
|  | Peeled dried roots |  |
|  | Petroleum ether or chloroform extracts |  |
|  | Powdered stem |  |
| Gum | Ethanolic extracts |  |
|  | Gum exudates |  |
| Flower | Aqueous extract of flowers |  |
|  | Chloroform extract of flowers |  |
|  | Ethanolic extracts |  |
|  | Ethyl acetate extract of flower |  |
|  | Flower powder |  |
|  | Fried flower |  |
| MO: *Moringa oleifera* | | |
